# Evaluation of initial chest computed tomography (CT) findings of COVID-19 pneumonia in 117 deceased patients: a retrospective study

**DOI:** 10.3906/sag-2009-183

**Published:** 2021-06-28

**Authors:** Yasemin GÜNDÜZ, Oguz KARABAY, Ali Fuat ERDEM, Erbil ARIK, Mehmet Halil ÖZTÜRK

**Affiliations:** 1 Department of Radiology, Faculty of Medicine, Sakarya University, Sakarya Turkey; 2 Department of Infectious Diseases and Clinical Microbiology, Faculty of Medicine, Sakarya University, Sakarya Turkey; 3 Department of Anesthesiology and Reanimation, Faculty of Medicine, Sakarya University, Sakarya Turkey

**Keywords:** Chest computed tomography, COVID-19 infection, mortality

## Abstract

**Background/aim:**

There is no study in the literature in which only chest computed tomography (CT) findings of deceased cases obtained at admission were examined, and the relationship between these findings and mortality was evaluated.

**Materials and methods:**

In this retrospective study, a total of 117 deceased patients with COVID-19 infection confirmed by positive polymerase chain reaction and undergone chest CT were enrolled. We evaluated initial chest CT findings and their relationship, location, prevalence, and the frequency with mortality.

**Results:**

The mean age of patients was 73 ±18 years; 71 of all patients were male and 46 were female. The predominant feature was pure ground-glass opacity (GGO) lesion (82.0%), and 59.8% of cases had pure consolidation. There was no cavitation or architectural distorsion. Pericardial effusion was found in 9.4% the patients, and pleural effusions were found in 15.3% of them. Mediastinal lymphadenopathy was only 11.9% in total.

**Conclusion:**

In deceased patients, on admission CTs, pure consolidation, pleural and pericardial effusion, mediastinal LAP were more common than ordinary cases. It was these findings that should also raise the concern when they were seen on chest CT; therefore, these radiologic features have the potential to represent prognostic imaging markers in patients with COVID-19 pneumonia.

## 1. Introduction

The rapid spread of the COVID-19 associated pneumonia and its urging mortality rate drew intense attention. The number of Corona virus cases has exceeded two million worldwide; as of September 10, 2020, the total number of confirmed cases is 27,738,179 worldwide with a reported mortality rate of 3.24% [1]. The first case was seen on 11 March 2020 in Turkey, the number of cases reached 28,455 as of 10 September 2020; 6895 patients (2.41 %) died.

Although most patients have mild symptoms and good prognosis, and sometimes disease progression can be rapid, COVID-19 can develop severe illnesses including pneumonia, pulmonary edema, acute severe respiratory distress syndrome, multiple organ failure, or it can even cause death [2,3]. Hence, accurate and early recognition of the disease is crucial not only for prompt implementation of treatment, but also for patient isolation and effective public health surveillance, containment and response [4]. Computed tomography (CT) is the major diagnostic radiologic tool in identifying the COVID-19 associated pneumonia among the suspected cases [5]. In our opinion, in addition to early diagnosis of the disease, earlier identification of tomography findings of cases with high risk of death can provide accurate information about the patient’s prognosis before the general condition of the patient deteriorates, thereby increasing the probability of recovery of the patient with an effective and timely treatment. This can only be achieved by evaluating the radiological findings of the deceased cases and by determining the findings that may be associated with mortality.

However, to the best of our knowledge, there is no study in the literature in which only chest CT findings of deceased cases obtained at admission were examined, and the relationship between these findings and mortality was evaluated. Therefore, in this study, we investigated the clinical and initial chest CT characteristics of 117 deceased COVID-19 cases obtained at admission and examined the association with mortality.

## 2. Materials and methods

### 2.1. Patients and groups

From March 15, 2020 to September 6, 2020, more than 8000 patients with suspected diagnosis of “viral pneumonia” underwent chest CT scanning in our institution. In this retrospective study, we included a total of 117 deceased patients with confirmed COVID-19 infection, who were hospitalized in Sakarya University Education and Research Hospital, and who met the inclusion criteria.

1- Patients with two positive results on real-time reverse transcriptase polymerase chain reaction (RT-PCR) testing for SARS-CoV-2 on endotracheal aspirate, nasopharyngeal swab, or oropharyngeal swab specimens.

2- Patients who underwent noncontrast-enhanced thin-section chest CT scans upon hospital admission (in first 12 h).

3- Age ≥ 18. 

4- Chest CT scan showing any evidence of pneumonia.

5- Patients with symptoms compatible with COVID-19 and no more than 5 days from onset.

6- Patients who have not recovered and died despite all kinds of treatment.

Currently, there is no specific treatment for COVID-19 with proven safety and effectiveness. According to the guidelines recommendations in our country, 200 mg Hydroxychloroquine tablet and/or 200 mg Favipiravir tablet are used for 5 days in our hospital. In addition, steroids and interleukin-6 blockers such as tosizumab are used in cases with macrophage activation. In addition, low molecular weight heparins were used in patients with thromboembolism risk. In patients whose clinical condition worsens despite medical treatment, intermittent or continuous oxygen inhalation with positive pressure in the presence of respiratory distress and intubation treatment are used. If it is not sufficient, specific treatment of accompanying organ failure is applied.

Clinical characteristics, laboratory findings, and epidemiologic data together with chest imaging manifestations of each confirmed cases were recorded.

All patients were clinically “moderate type”, and “in early phase” in terms of chest CT process when they were hospitalized.

Patients with a history of lung malignancy, lung surgery, tuberculosis or atelectasis, patients without clinical symptoms, patients in whom the date of the occurrence of the first symptom was unknown and patients having a normal chest CT scan, patients with severe artifacts on CT images, patients who were younger than 18 years old, and any of the other causes of pneumonia from common bacterial and viral pathogens were excluded from the study.

### 2.2. Chest CT scan

Chest CT imaging was performed on a Toshiba Aquilion CFX 64-detector CT scanner (Toshiba Medical Systems, Osaka, Japan). CT images were acquired at full inspiration with the patient in supine position and without administration of intravenous contrast medium. The scanning range was from the apex of lung to costophrenic angle. CT scan parameters are as follows: 64 slice slice of 0.5 mm with each 350 ms revolution, 13 cm coverage in patient axis direction, max scan range 180 cm, Gantry max clearance 180 cm, 70 kW HF generator with 400 ms scan speed. 

### 2.3. Review of CT images

We evaluated 19 major imaging features described based on Fleischner Society glossary of terms for thoracic imaging in previous studies [6]: ground-glass opacities (GGO), consolidation, crazy-paving pattern, air bronchogram, interlobular or intralobular septal thickening, centrilobular nodules, reverse halo/perilobular pattern, halo sign, architectural distortion, cavitation, tree-in-bud, bronchial wall thickening, reticulation, subpleural parenchymal bands, plevral thickening, traction bronchiectasis, intrathoracic lymph node enlargement (more than 1 cm in short-axis diameter), vascular enlargement in the lesion, pleural and/or pericardial effusions. 

Two senior radiologists (Y.G and M.H.O with 15 years of experience in interpreting chest CT imaging, respectively) evaluated the scanned images separately to identify chest CT characteristics of each patient. The focus was to observe whether lesions identified on the chest CT images involved both lungs and several lung lobes. A detailed analysis and evaluation of the imaging appearance concerning each of the lesions identified included: (i) the location and the number of lesions, (ii) the specific distribution of the lung lobe, (iii) lesion type.

Locations referred to different lobes and segments were involved. The distribution of the lesions was classified as: 1. upper, middle or lower zones, 2. peripheral or central 3. left, right or bilateral lungs. In this study, lung lobe distribution information included the right upper lobe (RUL), the right middle lobe (RML), the right lower lobe (RLL), the left upper lobe (LUL), and the left lower lobe (LLL). In addition, the distribution of the lung field included the peripheral or central zone and whether the peripheral and central zones were affected simultaneously (diffuse). The peripheral lung was defined as the outer one-third of the lung, and the central lung was defined as the inner two-thirds of the lung. The number of segments containing a specific category of opacities per patient were counted. 

The radiologist-defined lesion number and percentages of the whole lung, RUL, RML, RLL, LUL LLL were recorded, respectively. A computerized quantitative approach was used to evaluate the severity of COVID-19 in the whole lung, right lung, left lung, upper lobes, lower lobes, and each of the 5 lobes. 

The clinical parameters included age, sex, time from illness onset to hospital admission, comorbidities (systemic hypertension, diabetes mellitus, coronary heart disease, chronic renal disease, and chronic obstructive pulmonary disease), symptoms, clinical vital signs were collected and evaluated.

The main symptoms of patients detected at the first appication were fever, weakness, sore throat, dry cough, dyspnea, sputum, chest pain, fatique and myalgia, nause/vomiting, headache, respiratory rate (breathe/minute) heart rate (beat/minute). 

### 2.4. Statistical analysis

All statistical analyses were performed with SPSS software version 21.0 (SPSS Inc., Chicago, IL, USA) and P-values <0.05 were considered statistically significant. Continuous variables were expressed as median and compared with Student t-test or the Mann–Whitney U test); categorical variables were expressed as percentage and compared by χ2 test or Fisher’s exact test. In addition, the multivariate logistic regression analysis was performed to identify the relationship between mortality and initial CT features. 

Receiver operator characteristic (ROC) curve analysis was performed to calculate the threshold, specificity, sensitivity, and accuracy for the determination of possible factors (among clinical features and initial chest CT findings) on mortality.

## 3. Results 

### 3.1. Clinical characteristics

In the full cohort, the patients’ ages ranged from 28 to 95 years with a mean of 73 ± 18 years; 71 of all patients were male and 46 were female. 

The median length of hospital stay was 22±14 days, and the time of symptom onset before CT (range 0–5 days) was 3.9±1.6. 

The prevalence of hypertension was 37.6 % (n: 44) while chronic obstructive pulmonary disease, diabetes mellitus, chronic renal disease, coronary heart disease, malignancy, 33.3 % (n: 39), 11.9% (n:14), 6.8% (n:8), 16.2% (n:19), and 7.6% (n:9), respectively. 

The most common clinical symptoms were dyspnea (97/117, 82.9%), fever (93/117, 79.4%), sputum (88/117, 75.2%), dry cough (87/117, 74.3%), and weakness (85/117, 72.6%). In addition, mean respiratory rate of patients (breath/min) was 23 ± 10, and heart rate (beat/minute) was 85 ± 16. 

The demographic, clinical data and underlying disease, the rates of clinical symptoms, and vital signs of 117 patients at the time of admission has been shown in Table 1.

**Table 1:** The demographic and clinical data and underlying disease of 117 patients.

Characteristics	All (n:117)
Age (years)	73 ± 18 (28–95)
Sex (M/F)	71/46
Symptom onset before CT, (range, 0–5 days)	3.9 ± 1.6
The median length of hospital stay (day)	22±14
Coexisting comorbidities	(n,%)
COPD	39 (33.3%)
Diabetes	14 (11.9%)
Hypertension	44 (37.6%)
Chronic renal disease	8 (6.8%)
Coronary heart disease	19 (16.2%)
Malignancy	9 (7.6%)
Clinical symptoms/Vital signs	(n,%)
Fever	93 (79.4%)
Weakness	85 (72.6%)
Sore throat	44 (37.6%)
Dry cough	87 (74.3%)
Dyspnea	97 (82.9%)
Sputum	88 (75.2%)
Chest pain	48 (41.0%)
Fatigue or myalgia	83 (71.0%)
Gastrointestinal symptoms	36 (30.7%)
Headache	22 (18.8%)
Respiratory rate (breath/min)	23 ± 10
Heart rate (beat/min)	86 ± 16

Data reported as Mean ± SD or n (%) as appropriate.Abbrevations: COPD: Chronic Obstructive Pulmonary Disease, CT: Computerized Tomography.

### 3.1. Chest CT findings

**Figure 1:**
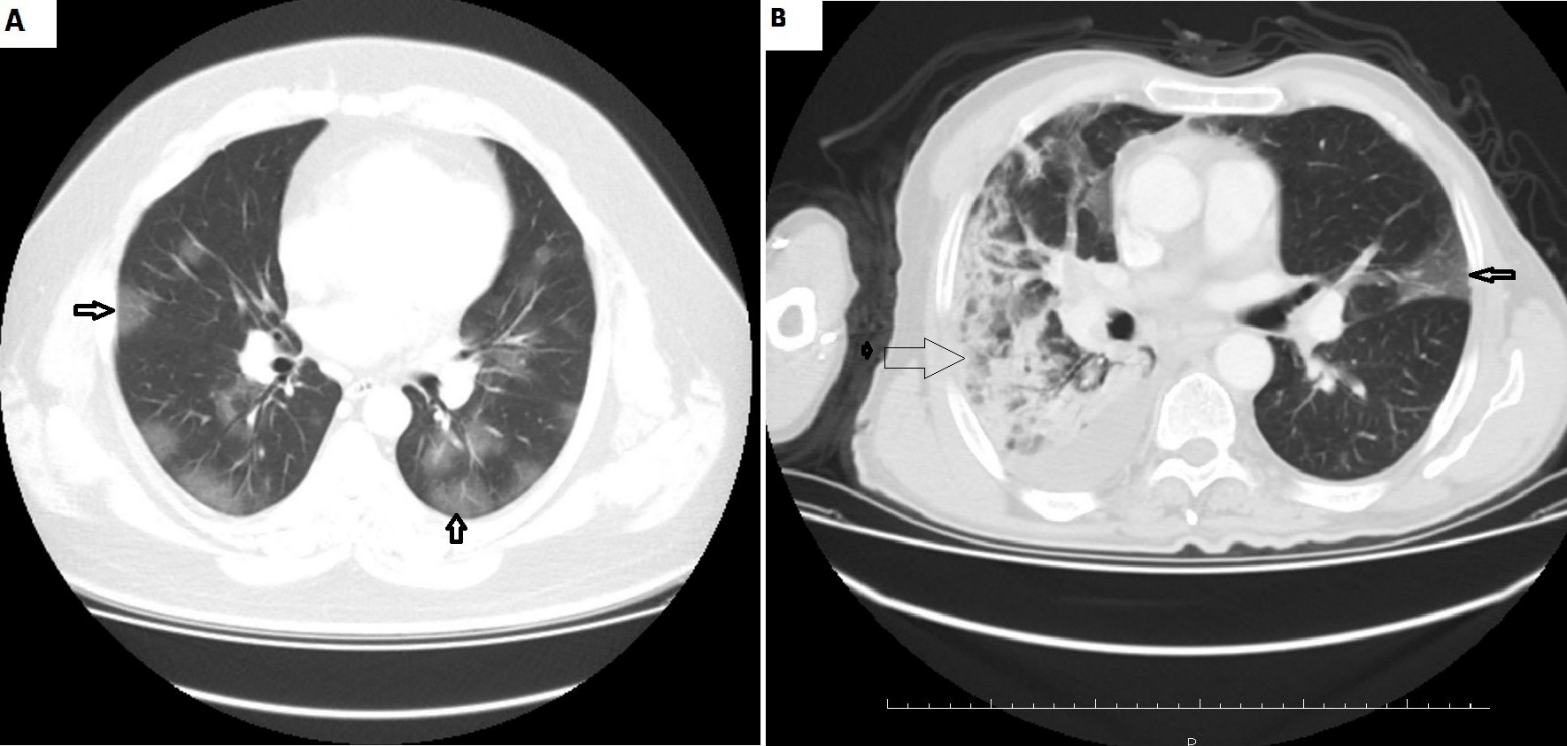
The presence of GG0 (A) and GGO together with widespread consolidation (B) in the axial lung window section of admission chest CT of these patients (1a: 64 years age, male, with hpertension; 1b: 43 years age, male) who lost their lives, draw attention. Thick little arrows: ground glass opacity; thin big arrow: consolidation.

The predominant feature was pure GGO lesion (96/117, 82.0%), and 59.8% (70/117) of cases had pure consolidation. In addition, 46 patients (39.3%) had mixed (GGO and consolidation) lesion (Figure 1a, 1b), and 6 patients (5.8%) had neither GGO nor consolidation. 

There was no cavitation or architectural distorsion in our cohort of cases. Halo sign (2/117, 1.7%), reverse halo sign (3/117, 2.5%), vascular enlargement (3/117, 2.5%), reticulations (4/117, 3.4%), and tree-in-bud nodularity (4/117, 3.4%) were also rare findings. In addition, crazy paving patern (5/117, 4.2%), centrilobular nodules (6/117, 5.1%), bronchial wall thickening (6/117, 5.1%), subpleural bands (7/117, 5.9%), air-bronchograms (9/117, 7.7%), and pleural thickenings (12/117, 10.2%) were other rare findings that which were detected. 

**Figure 2:**
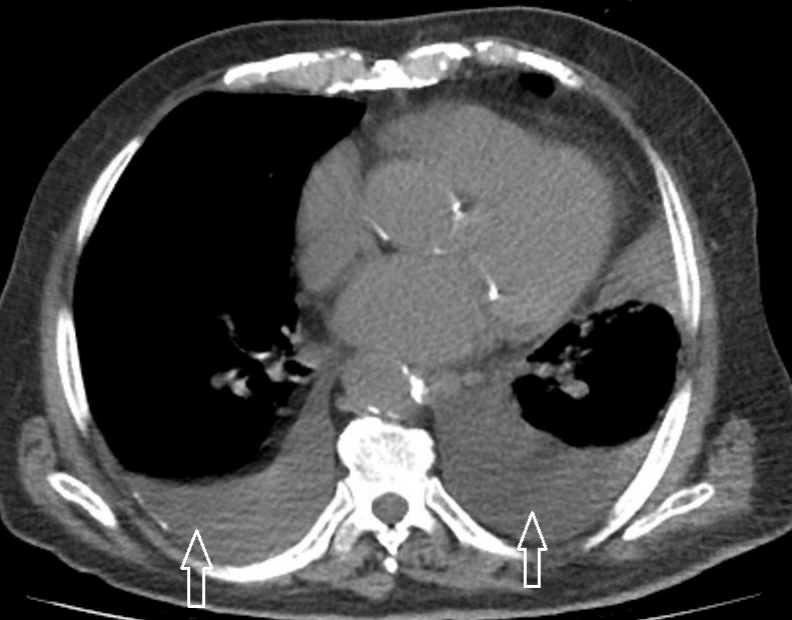
Significant pleural effusion was detected in the Chest CT of this patient (70 years age, male, with diabetes mellitus), and it was shown that the presence of pleural effusion was an important determinant of mortality in our patients over 65 years of age. White arrows: pleural effusion.

**Figure 3:**
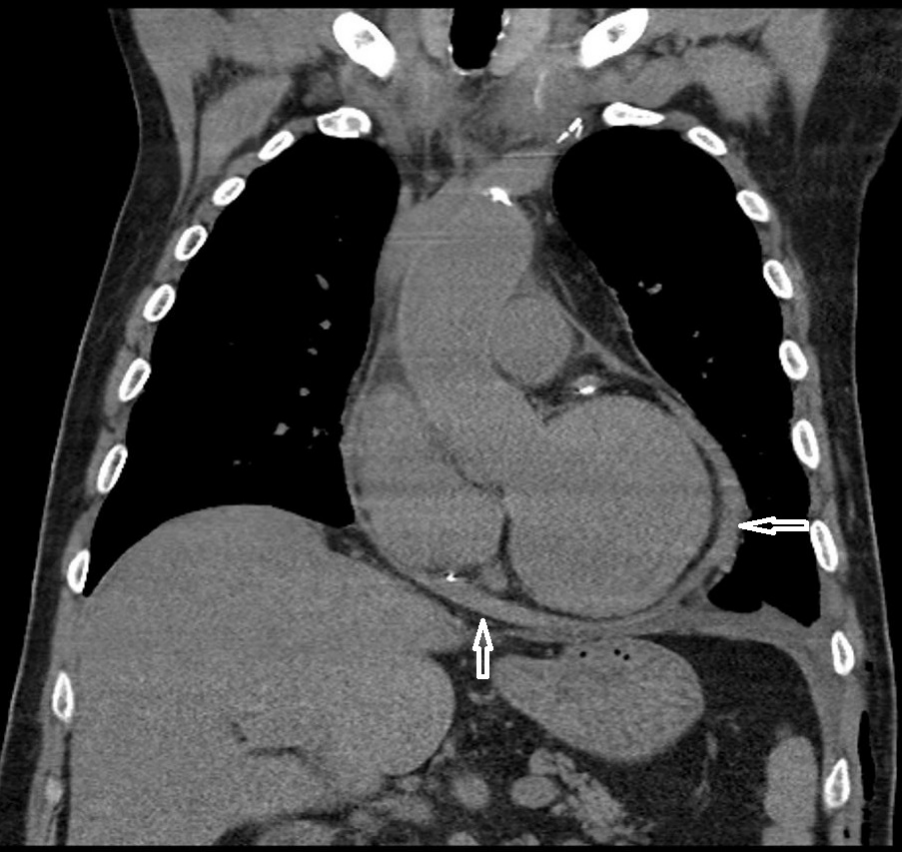
At the admission chest CTs of this case (58 years age, male, with hypertension and coronary artery disease), there was pericardial effusion in coronal section. This case also died despite all kinds of treatment. White arrows: pericardial effusion.

**Figure 4:**
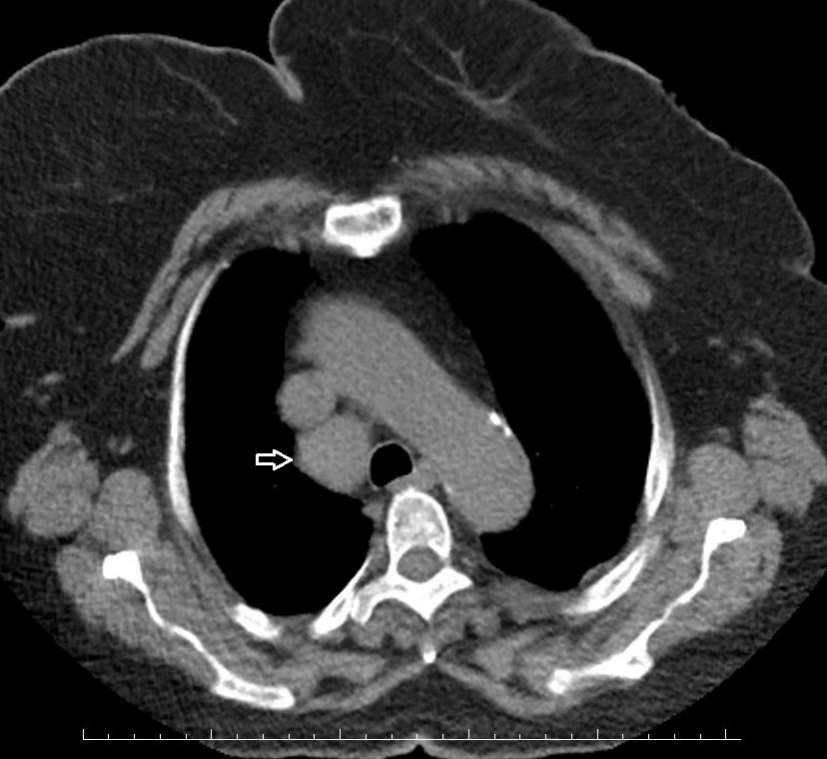
In the chest CT of the patient (64 years age, female, with diabetes mellitus and chronic renal disease), it was seen that there was marked right lower paratracheal (4R) lymphadenopathy in the noncontrast axial section. White arrow: lymphadenopathy.

Pericardial effusion was found in 11/117 (9.4%) of patients (Figure 2), and pleural effusions were found in 18/117 (15.3%) of patients (Figure 3), and the number of patients with mediastinal lymphadenopathy was only 14 (11.9%) in total (Figure 4).

The number of lung lesions on chest CT scans were counted for each infected patient and comprised a total of 1100 lesions, and CT score (number of lesion per patient) was 9.40.

A computerized quantitative approach was used to evaluate the prevalence of Chest CT lesions of COVID-19 infection, in the whole lung, right lung, left lung, and each of the 5 lobes. Of a total of 1100 lesions spread the whole the lung, 176 lesions (10.6%) were located in the RUL, 59 (5.3%) were in RML, 260 (23.6%) were in RLL, 237 (21.5%) were in LUL, 368 (33.4%) were in LLL. While the lobe with the least lesion involvement was RML, the lesion was mostly detected in LLL.

The chest CT features, including the location, extent and distribution of lesions were summarized in Table 2.

**Table 2:** The chest CT findings of 117 patients at the time of admission.

Chest CT findings	All (n:117)	Chest CT findings	All (n:117)
Ground glass opacities (GGO)	96 (82.0%)	Vascular enlargement	3 (2.5%)
Consolidation	70 (59.8%)	Architectural distortion	0 (0%)
Mixed (GGO and consolidation)	46 (39.3%)	Tree-in-bud nodularity	4 (3.4%)
Neither GGO nor consolidation	6 (5.8%)	Bronchial wall thickening	6 (5.1%)
Halo sign	2 (1.7%)	Crazy paving pattern	5 (4.2%)
Reverse halo/perilobular pattern	3 (2.5%)	Pleural effusion	18 (15.3%)
Centrilobular nodules	6 (5.1%)	Pericardial effusion	11 (9.4%)
Plevral thickening	12 (10.2%)	Mediastinal lymphadenopathy	14 (11.9%)
Reticulation	4 (3.4%)	Septal thickening interlobuler	16 (13.6%)
Air bronchograms	9 (7.7%)	Septal thickening intralobuler	15 (12.8%)
Subpleural bands	7 (5.9%)	Bronchiectasis	22 (18.8%)
Cavitation	0 (0%)	Emphysema	17 (14.5%)
Transverse distribution	(n/%)	Unilateral involvement	7 (6.0%)
Central	6 (5.2%)	Bilateral involvement	110 (94.0%)
Peripheral	53 (45.3%)	Total number of lesions	1100
Diffuse (central+peripheral)	58 (49.5%)	CT score (lesion number/n)	9.40
Lobar location	Lesion number	Symptom onset before CT, (range, 0–5 days)	3.9 ± 1.6
Right upper lobe (RUL)	176	1 lobe involvement (n/%)	8 (6.8%)
Right middle lobe (RML)	59	2 lobe involvement (n/%)	14 (12%)
Right lower lobe (RLL)	260	3 lobe involvement (n/%)	20 (17%)
Left upper lobe (LUL)	237	4 lobe involvement (n/%)	34 (29%)
Left lower lobe (LLL)	368	5 lobe involvement (n/%)	41 (35%)

When the effect of age on lesion distribution was evaluated by dividing the patients into two groups as those over 65 years old (n:69) and those aged 65 and under (n:48), only pure consolidation (48 patients, 55.0% vs 22 patients, 45.8%, P: 0.004) and the number of patients with pleural effusion (14 patients, 20.2% vs 4 patients, 8.3%, P: 0.011) were statistically different, and these two tomography findings were significantly more pronounced in patients over 65 years of age. There was no significant difference in other findings (P > 0.05).

Comparison of chest CT findings of the patient group >65 years of age and the group ≤ 65 years of age was shown in Table 3. 

**Table 3:** Comparison of chest CT findings of the patient group >65 years of age and the group ≤ 65 years of age.

Chest CT findings	Age ≤ 65 (28–65)(n: 48)	Age > 65 (66–95)(n: 69)	P value
Ground glass opacities (GGO)	39 (81.2%)	57 (82.6%)	0.781
Consolidation	22 (45.8%)	48 (69.5%)	0.004
Mixed (GGO and consolidation)	20 (41.6%)	26 (37.6%)	0.320
Neither GGO nor consolidation	2 (4.1%)	4 (5.7%)	0.632
Halo sign	0 (0%)	2 (2,8%)	0.199
Reverse halo/perilobular pattern	1 (2.0%)	2 (2.8%)	0.704
Centrilobular nodules	3 (6.2%)	3 (4.3%)	0.298
Plevral thickening	5 (10.4%)	7 (10.1%)	0.933
Reticulation	2 (4.1%)	2 (2.8%)	0.714
Air bronchograms	4 (8.3%)	5 (7.2%)	0.612
Subpleural bands	2 (4.1%)	5 (7.2%)	0.084
Cavitation	0 (0%)	0 (0%)	------
Vascular enlargement	1 (2.0%)	2 (2.9%)	0.771
Architectural distortion	0 (0%)	0 (0%)	------
Tree-in-Bud nodularity	2 (4.1%)	2 (2,8%)	0.489
Bronchial wall thickening	3 (6.2%)	3 (4.3%)	0.117
Pleural effusion	4 (8.3%)	14 (20.2%)	0.011
Pericardial effusion	4 (8.3%)	7 (10.1%)	0.232
Mediastinal lymphadenopathy	5 (10.4%)	9 (13.0%)	0.318
Crazy paving pattern	3 (6.2%)	2 (2.9%)	0.059
Septal thickening interlobulerSeptal thickening intralobuler	7 (14.5%)6 (12.5%)	9 (13.0%)9 (13.0%)	0.7110.815
Bronchiectasis	8 (16.6%)	14 (20.2%)	0.664
Emphysema	7 (% 14.58)	10 (14.49%)	0.992
Total number of lesions	522	578	0.196
CT score (lesion number/n)	9.52	9.28	0.750
Symptom onset before CT, (range, 0–5 days)	3.8±1.4	4.0±1.7	0.213
The median length of hospital stay (day)	20±13	24±16	0.717

In our cases, advanced age (especially over 65 years), high CT score, presence of consolidation, pleural effusion, pericardial effusion, and mediastinal lymphadenopathy were noted to be higher than usual. With multivariate logistic regression analysis, the relationship of each with mortality was examined, and, in this analysis, advanced age (> 65 years), pleural effusion, and the presence of consolidation were found to be statistically significant factors associated with mortality. The results of multivariate logistic regression analyzes were shown in Table 4.

**Table 4:** Multivariable logistic regression analysis for predicting of the mortality in 117 patients hospitalized with COVID-19.

Characteristics	Odds ratio	95% CI	P value
Age (> 65 years)	3.53	1.17–6.11	0.01
Pleural effusion	4.94	1.01–10.82	<0.001
Consolidation	1.33	1.09–1.68	0.03

In addition, for ROC analysis, the area under the ROC curve (AUC) of pleural effusion was 0.92 (95% CI:0.83-0.97; P<0.001) for the prediction of poor prognosis and mortality. When the cutoff value of age was 65 years, the sensitivity and specificity were 93.7% and 85.9%, respectively. In addition, AUC was found 0.80 (95% CI:0.71–090; P : 0.022) for consolidation and the sensitivity and specificity were 77.7% and 69.5%, respectively. 

## 4. Discussion

The number and prevalence of tomography lesions that occur from the onset of symptoms in COVID-19 pneumonia increase and/or change day by day and at every stage, depending on the positive or negative course of the disease [7]. Therefore, there are group differences of some CT signs among different CT stages, though GGOs and consolidation were most frequently seen in each CT stages without group differences in patients with COVID-19 pneumonia [8]. However, in our study, chest tomographies of all cases were taken within the first 5 days when their symptoms started (in the early stage as a CT stage, in the moderate stage as a clinical stage), and were not taken again later or were not evaluated even if they were taken. 

In our cases, the predominant abnormal chest CT patterns observed were bilateral, multilobar, diffuse (central plus peripheral), and infiltrated the lower lobes GGOs; however, consolidations, pericardial and pleural effusion, mediastinal lymphadenopathy occured more often than avarage of usual cases [9]. In the literature, from the ordinary stage to the severe/critical stage, in more cases, the number of involved lung segments and lobes, the frequencies of consolidation, intra/interlobular septal thickening, crazy-paving pattern, and air bronchogram all increased [10]. However, in our study, unlike the critical case features mentioned previously, crazy-paving pattern and air bronchogram were much less common, and extrapulmonary findings of pleural and pericardial effusion and lymphadenopathy (which have been shown to be poor prognostic indicators in cases of viral pneumonia) were seen more frequently on initial tomography [11].

Pan et al. classified CT findings of COVID-19 according to 4 temporal stages as early, rapid progressive, peak (consolidation), and absorption (dissipation) stages [7]. Our cases were symptomatic and since chest CTs were taken within the first 5 days after the onset of symptoms, they were expected to show radiological findings of early stage or early period of rapid progressive stage. Each CT stage has its characteristic CT signs and performances, making it possible for radiologists and physicians to quickly determine the stage of the pneumonia [12]. However, what is important is to determine the clinical and radiological findings that may be associated with high mortality before the patient’s general condition deteriorates and reaches the critical stage, for predicting the prognosis and outcome, taking necessary preventive measures, especially in high-risk cases, and ensuring early treatment of the patient, thus preventing or reducing mortality.

As a matter of fact, in the early stage (days 1–4 of symptomatic presentation), chest CT findings include single or multiple GGOs distributed subpleurally in the lower lobes unilaterally or bilaterally, or GGO combined with interlobular septal thickening, patchy consolidations in ordinary cases. In addition, interlobular septal thickening, air bronchus sign, intralesional and/or perilesional bronchiectasis, and bronchial wall thickening were less seen than in the progressive stage. The reticular sign, pleural thickening, and interlobar fissure displacement were not common, and the frequency of pleural effusion, pericardial effusion, and mediastinal lymphadenopathy was relatively small in early stage. In the rapid progressive stage (days 5–7 of symptomatic presentation), the infection exacerbates rapidly and expands with a bilateral multilobe distribution and diffuse GGO, crazy paving pattern, air bronchograms and large, light consolidative opacities are formed [7,12–14].

In a study, patients with mild form of disease showed pure GGO more often than patients with severe disease; on the contrary, pure consolidation was more common in patients with severe disease. Thus, it could be speculated that pure GGO might be associated with early or mild stage of disease; on the contrary, pure consolidation might show severe clinical form or be seen at a more advanced stage [13-16]. In another study, on admission, GGO, consolidation were the major CT fndings in both groups, bilateral lung involvement, difuse distribution were more common in nonsurvivors than survivors [16]. Considering the presence of consolidation together with GGO, which was seen as an early finding in our cases, although GGO was seen in usual rate as a typical finding, consolidation was more common than avarage of usual [8]. It was noteworthy that there were ≥3 lobes involved in 4/5 of the cases, and 4-5 lobes were involved in 2/3 of the cases.

The rate of patients with interlobular septal thickening and air bronchogram was higher in patients with severe disease than in patients with mild disease, indicating that these two findings could relate to advanced or late stage of COVID-19 pneumonia or coexistence of superimposed processes, such as pulmonary edema [10,18]. In addition, it was stated that severe and widespread involvement increases the frequency of architectural distortion, and bronchiectasis is seen more frequently as a concomitant disease [18,19]. Likewise, in our study, it was found that both bronchiectasis and emphysema were coomon underlying disease. However, in contrast, the fact that air bronchogram and arcitectural distortion were not encountered frequently in our study suggests that these findings were not seen in the early period, that is, they may not be seen in the tomographies taken within the first 5 days. Wang et al.  investigated changes of CT findings in COVID-19 patients, and they found that the extent of pulmonary involvement peaked during days 6–11 after the onset of initial symptoms [20]. 

It is common to see the crazy paving pattern in chest CTs taken 8–9 days after the onset of symptoms. However, increasing alveolar edema, exudate lymphocyte infiltrations, accumulation in the interstitial space, radiologically, the formation of crazy paving pattern, and the increase in the frequency of this appearance indicate the progress of the disease, and a poor prognosis [21–23]. In our cases, since the tomographies were taken within the first 5 days of the onset of symptoms, the pathophysiological process that would lead to an increase in the frequency of the crazy paving pattern has not yet settled, so this finding indicating a poor prognosis is not common in the first 5 days. Similar evaluation can be made for the development of air bronchogram, and the increase in air bronchogram frequency is again a sign of severe/advanced disease and shows poor prognosis [22], but this finding is not a common finding in CTs taken within the first 5 days in patients who died.

Besides this, it has been showed that severe/critical patients have higher incidences of lymph node enlargement, pericardial effusion, and pleural effusion than ordinary patients [9,11]. Lymphadenopathy was usually rare in mild form of viral pneumonias, it was reported in 4%–8% of patients with COVID-19 [22]. Moreover, lymphadenopathy was considered one of the significant risk factors of severe/critical COVID-19 pneumonia [14]. Pleural and pericardial effusion were uncommon, manifesting mainly in patients with severe disease, which may indicate parapneumonic effusion or fluid overload [24]. We found more mediastinal lymphadenopathy (11.9%), pleural (15.3%) and pericardial effusion (9.4%) than usual, on the admission CT of our deceased patients [9], and we found that pleural effusion was a strong predictor of mortality, especially in patients over 65 years of age. These extrapulmonary lesions might indicate the occurrence of severe inflammation. In the presence of these findings, it is suggested that the cases carry a high risk for mortality and that we must take precautions as soon as possible.

In a study, older patients (>60 years) tended to have more areas of lung involvement with more lobes affected and more pleural thickening [25]. In another study, patients older than 50 years had more extensive disease with more consolidation as compared to patients younger than 50 years old. They tended to have more architectural distortion, bronchiectasis, mediastinal and hilar lymphadenopathy, pleural effusions, and worse outcomes than younger patients [9]. 

In accordance with the previous studies, in a different study, old patients (63–78 years) with more comorbidities such as hypertension, diabetes, and coronary heart disease were more inclined to develop fatal ARDS in nonsurvivor group [26]. Our patient sample was different from those in other studies as we evaluated the clinical and imaging features in the deceased cases with COVID-19. Since emphysema and bronchiectasis (14.5% and 18.8%) as underlying dieases were common in the deceased patients, it could be hypothesized that an underlying lung disease might also cause worse clinical outcome. Furthermore, patients with hypertension (37.6%), and the old patients (73±18) appeared more frequently in the deceased cases. These findings suggested that 2019-nCoV was more likely to infect elderly people with chronic comorbidities as a result of the weaker immune functions of these patients, and 2019-nCoV-associated death was also related to elder age and underlying illnesses, especially hypertension, cardiac, and pulmonary disease.

In a previous study, patients in deceased group were much older than survivors, and logistic regression analysis revealed that age ≥ 65 years is a strong predictor for death of COVID-19 pneumonia. Actually, in the whole cohort of 179 COVID-19 pneumonia patients, no one died who was younger than 50 years, whereas 17 (81%) of deceased patients were older than 65 years [27]. However, in our study, there were 48 cases under the age of 65, 11 cases under the age of 50, and the youngest patient who died was 28 years old. As such, the number of cases in our study was considerably higher than the case number of this study, and includes younger patients. As a matter of fact, although many findings were similar in number and characteristics in cases under 65 years of age compared to cases above 65 years of age, halo sign, subpleural band, pericardial effusion, mediastinal LAP and brochiectasis tended to be more, whereas the number of cases with pleural effusion (4 vs 14, P: 0.011) and pure consolidation number (45.8% vs 69.5%, P: 0.004) were statistically significantly higher. In the ROC analysis, it was seen that pleural effusion had high sensitivity and specificity as a mortality predictor in cases above 65 years of age, especially when the age of 65 was cutoff, and the detection of pleural effusion and pure consolidation in chest CT on hospital admission drew attention as a high mortality predictor, especially in patients over 65 years of age.

In deceased patients, on admission CT, extensive, bilateral, multilobar, basal, both central and peripheral GGO and consolidative opacities, without cavitation and architectural distortion were occured. In addition, rarely air bronchogram, pleural and bronchial wall thickening, crazy paving pattern, subpleural bands, centrilobular nodules, pure consolidation, pleural and pericardial effusion, mediastinal LAP, and emphysema and very rarely halo sign, reverse halo sign, reticulation, vascular enlargement, were detected. It was these findings that should also raise the concern when they were seen on chest CT; therefore, these radiologic features have the potential to represent prognostic imaging markers in patients with COVID-19 pneumonia. In the previous studies, the radiological findings of the patients who died were compared with those who survived, and comments were made based on the findings obtained [16,17]. However, in our study, only the CT findings of the deceased cases taken at the time of admission were evaluated retrospectively, and only these CT findings were interpreted without causing confusion. Therefore, there are not many similar studies in the literature or its methodology is quite different from studies considered to be similar.

There are a few limitations to our study. First **,** the sample size was relatively small owing to the strict selection criteria applied to these patients, only 117 patients with confirmed COVID-19 were included. Although it is probably one of the studies with the highest number of patients in which only deceased patients were recruited, it is not definitive, studies containing more cases are needed to prevent death with the patient, but we underline that this is not desired. A more comprehensive approach could be provided to make the good or poor prognosis predictable from the initial chest CT findings. However, the desire and effort of publishing the valuable radiological findings obtained in a pandemic as soon as possible to be beneficial to the public health can be defined as another underlying factor of this limitation. 

Second **,** we focused on baseline CT findings that clinicians and radiologists first encountered, rather than findings from follow-up CT scans. Thus, we did not apply control CT to our patients and did not evaluate what was done. In order to evaluate changes in lung parenchyma that may develop over time during the course of the disease, taking and evaluating lung CT for control or follow-up in patients was out of the protocol of our study and we did not apply this method for many studies. In the future, studies with larger patient groups and longer follow-up may be performed to evaluate the prognostic value of the first CT features. 

Third, in our study, we did not evaluate the role of chest CT in monitoring response to treatment. 

Fourth, no autopsy was performed in the deceased patient group. Upon comparison with the pathological results, additional and a more precise interpretation of the CT image signs will be available in the future work.

Lastly, this was a retrospective study with initial CT images during hospitalization, mainly demonstrated the early phase of chest CT lesions in patients with COVID-19.

## Informed consent

This retrospective study was approved by the Ethics Committee of Sakarya University Faculty of Medicine. Approved Number:71522473/050.01.04/479 and the requirement for informed consent was waived by the ethics committee. 
